# Improvement in the outcomes of mantle cell lymphoma in the last decade: a real-life non interventional study of the Croatian Cooperative Group for Hematologic Diseases

**DOI:** 10.3325/cmj.2021.62.455-63

**Published:** 2021-10

**Authors:** Sandra Bašić-Kinda, Karla Mišura Jakobac, Jasminka Sinčić-Petričević, Dajana Deak, Marijo Vodanović, Marinka Jakić-Bubalo, Zdravko Mitrović, Aron Grubešić, Barbara Dreta, Dubravka Županić Krmek, Božena Coha, Delfa Radić-Krišto, Igor Aurer

**Affiliations:** 1 Division of Hematology, Department of Internal Medicine, University Hospital Center Zagreb, University of Zagreb, School of Medicine, Zagreb, Croatia; 2 Division of Hematology, Department of Internal Medicine, University Hospital Merkur, Zagreb, Croatia; 3 Department of Hematology, Clinic of Internal Medicine, University Hospital Center, Osijek, Croatia; 4 Division of Hematology, Department of Internal Medicine, Sestre Milosrdnice University Hospital Center, Zagreb, Croatia; 5 Division of Hematology, Department of Internal Medicine, University Hospital Center Split, Split, Croatia; 6 Department of Internal Medicine, University of Zagreb, School of Medicine, Zagreb, Croatia; 7 Division of Hematology, Department of Internal Medicine, University Hospital Center Rijeka, Rijeka, Croatia; 8 Department of Internal Medicine, Sveti Duh University Hospital Center, Zagreb, Croatia; 9 Department of Internal Medicine, Dr. Josip Benčević General Hospital, Slavonski Brod, Croatia

## Abstract

**Aim:**

To compare the outcomes of Croatian patients with mantle cell lymphoma (MCL) who started treatment in 2007 and 2008 (historical cohort) and of those who started treatment between 2015 and 2017 (recent cohort).

**Methods:**

The historical cohort consisted of 40 patients who started treatment with rituximab in 2007 and 2008. Data on the recent cohort, consisting of 89 patients, were collected retrospectively from the electronic databases of Croatian hospitals with hematology units. Demographic characteristics and data on induction regimens, autologous stem cell transplantation (ASCT), and rituximab maintenance in the first remission, event-free survival (EFS), and overall survival (OS) were available for both cohorts, and data on cell morphology, mantle cell international prognostic index (MIPI), and Ki67 expression only for the recent cohort.

**Results:**

The recent cohort had significantly better two-year EFS and OS (EFS 58% vs 40%, *P* = 0.014; OS 80% vs 56%, *P* = 0.009), especially in patients below 65. In univariate analysis, induction regimen, ASCT, and maintenance were significant prognostic factors for EFS and the former two for OS. In the multivariate analysis, only ASCT remained significant. Bendamustine + rituximab (BR) induction improved the outcomes of non-transplantable patients over R-CHOP R-CHOP (rituximab, cyclophosphamide, doxorubicin, vincristine, steroid). Blastoid morphology and high MIPI were adverse prognostic factors for EFS and OS.

**Conclusion:**

In the last decade, the outcome of newly diagnosed MCL patients improved. ASCT in the first remission was the main contributor in transplantable patients and BR in non-transplantable. Regularly updated national guidelines may help in a timely adoption of new treatments, thus improving the results.

^10^Division of Hematology, Department of Internal Medicine, University Hospital Dubrava, Zagreb, Croatia 

^11^Medical School, University of Osijek, Osijek, Croatia

Mantle cell lymphoma (MCL) is a rare type of B-cell non-Hodgkin lymphoma (B-NHL), comprising 3%-10% of all cases (1,2). At presentation, the disease is usually disseminated, with a progressive course and a continuous tendency to relapse. An indolent variant has recently been identified. MCL is more frequent in men. Adverse prognostic factors include high mantle-cell international prognostic index (MIPI – age, performance status, LDH, leukocyte count) (3), high Ki67 expression, and blastoid morphology (4). The median overall survival (OS) increased in the last decade from around 3 to more than 5 years, corresponding to the introduction of high-dose cytarabine (HD-AraC) and bendamustine into front-line induction therapy, autologous stem cell transplantation (ASCT) in the first remission, and rituximab maintenance, but the individual impact of each of these factors is unclear (5).

The Croatian Cooperative Group for Hematologic Diseases (KroHem) performed this retrospective non-interventional real-life study to help elucidate factors that contributed to the observed improvement. Data on the characteristics, treatment, and outcomes collected from patients with MCL who were diagnosed or started treatment between 2015 and 2017 (recent cohort) were collected retrospectively and compared with those of patients starting treatment with a rituximab-containing regimen in 2007 and 2008 (historical cohort).

## PATIENTS AND METHODS

The historical cohort consisted of patients with B-NHL who started front-line treatment with rituximab in 2007 and 2008. The data were obtained from Croatian hematology centers and hematologists. Information on demographic characteristics, front-line treatment, response to therapy, event-free survival (EFS), and OS were collected. The recent cohort was identified retrospectively from hospitals' electronic databases and included all patients with MCL who started treatment or were diagnosed and not treated between January 1, 2015 and December 31, 2017. Hematologists from all Croatian hospitals with hematology units participated in the study. Information on demographic characteristics, MIPI, Ki67, morphology (classical vs blastoid), front-line treatment, response to therapy, EFS, and OS were collected. EFS was defined as the time from treatment start to the first of the following: failure to achieve remission with front-line therapy, relapse after achieving remission, or death of any cause. OS was defined as the time from treatment start to death of any cause. All included patients were previously untreated. The study was approved by the Ethics Committee of the University Hospital Centre Zagreb.

### Statistical analysis

All analyses were performed using data from treated patients. EFS and OS curves were generated according to the Kaplan-Meier method. Univariate analysis was performed with the log-rank test using an Excel-based computer program developed by a member of KroHem (6). Multivariate analysis was performed using SPSS, version 21 (IBM, Armonk, NY, USA). The *P* values below 0.05 were considered statistically significant. Since patients who fail to respond to induction treatment or relapse early do not continue with ASCT and/or maintenance, to avoid bias only patients with an EFS of at least 6 months were included in the analyses of the effect of ASCT and maintenance.

## RESULTS

### Historical control

The historical cohort consisted of 40 patients, 28 men (70%), with a median age of 67 years (Table 1). Data on patients who were not treated or did not receive rituximab as part of their front-line regimen were not collected. The median follow-up was 39 months. Thirty-six patients were treated with R-CHOP (rituximab, cyclophosphamide, doxorubicin, vincristine, steroid) and 4 with R-CVP (rituximab, cyclophosphamide, vincristine, steroid). Thirteen percent of the patients with an EFS of six months or longer were autografted in the first remission. None received bendamustine, HD-AraC, or maintenance.

**Table 1:** Characteristics of patients who started treatment in 2007 and 2008 (historical cohort) and of those who started treatment between 2015 and 2017 (recent cohort)

	Historical cohort	Recent cohort
Number of patients	40	82
Age in years; median (range)	67 (44-82)	66 (35-90)
Sex; number (%)		
male	28 (70)	57 (70)
female	12 (30)	25 (30)
Median follow-up in months	39	20
White blood count (median/range)	Data not available	8.6/1.0-86.7 × 10^9^/L
ECOG performance status; number (%)		
0-1	68 (83)	29 (73)
2-4	14 (17)	11 (28)
Lactate dehydrogenase; number (%)		
within the reference range	47 (57)	24 (60)
above the reference range	35 (43)	16 (40)
Time from diagnosis to treatment in months; median (range)	1 (1-17)	1 (1-46)
Induction regimen; number (%)		
R-CVP	4 (10)	0
R-CHOP-like	36 (90)	29 (35)
BR	0	22 (27)
HD-AraC containing	0	31 (38)
ASCT, number (%)^†^		
yes	5 (15)	24 (35)
no	28 (85)	44 (65)
Rituximab maintenance, number (%)^†^		
yes	0	31 (46)
no	33 (100)	37 (54)
Event-free survival at two years, %	58	40
Overall survival at two years, %	80	56

*Abbreviations: R-CVP – rituximab, cyclophosphamide, vincristine, steroid; R-CHOP – R-CVP + doxorubicine; BR – bendamustine, rituximab; HD-AraC – high-dose cytarabine; ASCT – autologous stem cell transplantation in the first remission.

†only patients with EFS≥6 months.

### Recent cohort

The recent cohort consisted of 89 patients, 60 men (67%), with a median age of 67 years. The median follow-up was 20 months. Seven patients were not treated, 5 had indolent disease, and 2 frail elderly patients opted for best supportive care only. In order to make the two groups as comparable as possible, only the outcomes of treated patients were used for comparisons. Of the 82 treated patients, 22 received bendamustine + rituximab (BR), 29 R-CHOP, 25 R-CHOP alternating with R-DHAP (rituximab, dexamethasone, HD-AraC, cisplatin), 1 R-CHOP alternating with HD-AraC, and 5 R-BAC (rituximab, bendamustine, HD-AraC). For the purpose of this analysis, the latter three regimens were grouped together as HD-AraC-containing regimens. No patient received R-CVP. Thirty-five percent of patients with an EFS of six months or longer were autografted in the first remission, while 48% received rituximab maintenance.

### Survival

**Figure 1:**
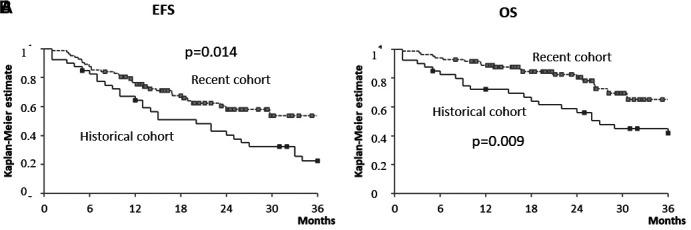
Event-free survival (**A**) and overall survival (OS) (**B**) according to the time of treatment. Full line – historical cohort; dashed line – recent cohort.

None of the 5 patients with indolent MCL needed treatment. The two-year EFS of treated patients improved from 40% to 58% (*P* = 0.014) and two-year OS from 56% to 80% (*P* = 0.009) (Figure 1). The outcomes in patients younger than 65 significantly improved (two-year EFS 47% vs 75%, *P* = 0.004; two-year OS 54% vs 92%, *P* = 0.005). The difference in the outcomes of patients older than 65 was not significant (two-year EFS 36% vs 41%, *P* = 0.674; two-year OS 57% vs 70%, *P* = 0.368).

### Effect of different therapeutic modalities

**Figure 2:**
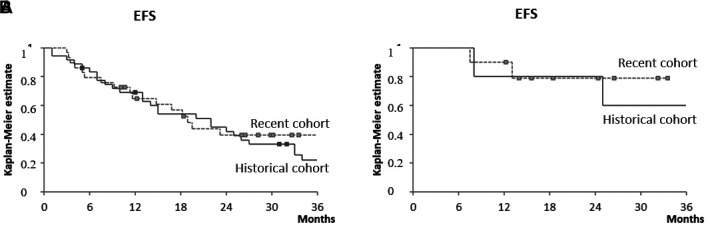
Event-free survival (EFS) of patients treated with R-CHOP (rituximab, cyclophosphamide, doxorubicin, vincristine, steroid) without maintenance (**A**), EFS of patients treated with ASCT without maintenance (**B**). Full line – historical cohort; dashed line – recent cohort.

The EFS curves of patients from the historical and recent cohort receiving the same treatments overlapped (Figure 2). We therefore analyzed the effect of different therapeutic modalities in all 122 patients.

**Figure 3:**
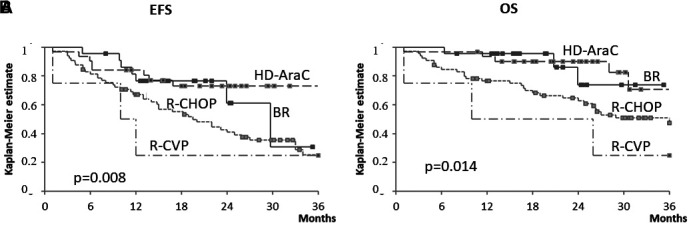
Event-free survival (EFS) (**A**) and overall survival (OS) (**B**) according to the induction regimen.

**Figure 4:**
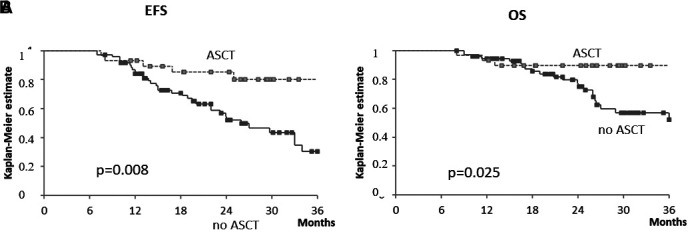
Event-free survival (EFS) (**A**) and overall survival (OS) (**B**) of patients alive and in remission for at least six months according to autologous stem cell transplantation in the first remission (ASCT). Full line – no ASCT; dashed line – ASCT.

**Figure 5:**
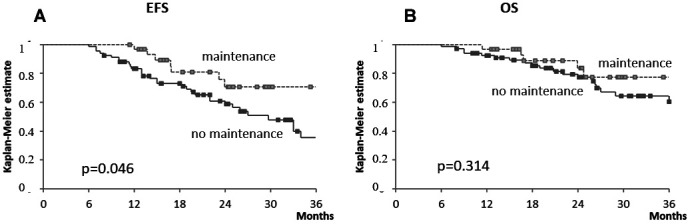
Event-free survival (EFS) (**A**) and overall survival (OS) (**B**) of patients alive and in remission for at least six months according to rituximab maintenance. Full line – no maintenance; dashed line – maintenance.

In the univariate analysis, induction regimen significantly influenced EFS (*P* = 0.008) and OS (*P* = 0.014) (Figure 3). Patients treated with HD-AraC-containing regimens had the best outcomes, followed by those treated with BR, R-CHOP, and R-CVP. The differences between the first and the last two regimens were significant. ASCT in the first remission significantly improved EFS (*P* = 0.008) and OS (*P* = 0.025) (Figure 4). Maintenance significantly improved EFS (*P* = 0.046), but not OS (*P* = 0.314) (Figure 5). In the multivariate analysis, ASCT remained the only significant prognostic factor for both EFS (*P* = 0.037) and OS (*P* = 0.024).

**Figure 6:**
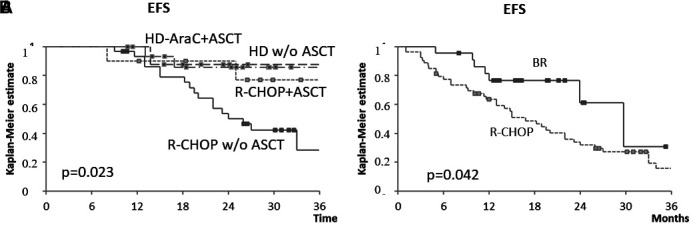
Event-free survival (EFS) of patients alive and in remission for at least six months treated with R-CHOP (rituximab, cyclophosphamide, doxorubicin, vincristine, steroid) or high-dose cytarabine-containing regimens (**A**) according to autologous stem cell transplantation in the first remission. EFS of untransplanted patients (**B**) according to induction regimen. Full line – BR; dashed line – R-CHOP; w/o – without.

Since the use of induction regimens differs between transplantable and non-transplantable patients, we analyzed the outcomes of different combinations of induction regimens, ASCT, and maintenance. None of the patients treated with BR out of 20 patients with an EFS of at least 6 months was autografted in the first remission, in comparison with 12 out of 51 patients treated with R-CHOP and 17 out of 26 patients treated with HD-AraC-containing regimens. The differences in the outcomes between transplanted and non-transplanted patients were pronounced in those receiving R-CHOP despite not reaching statistical significance, but not in those receiving HD-AraC. Untransplanted patients receiving BR had better outcomes than those receiving R-CHOP (*P* = 0.045) (Figure 6). The effect of rituximab maintenance was similar irrespective of the induction regimen (data not shown).

### Biologic prognostic factors

**Figure 7:**
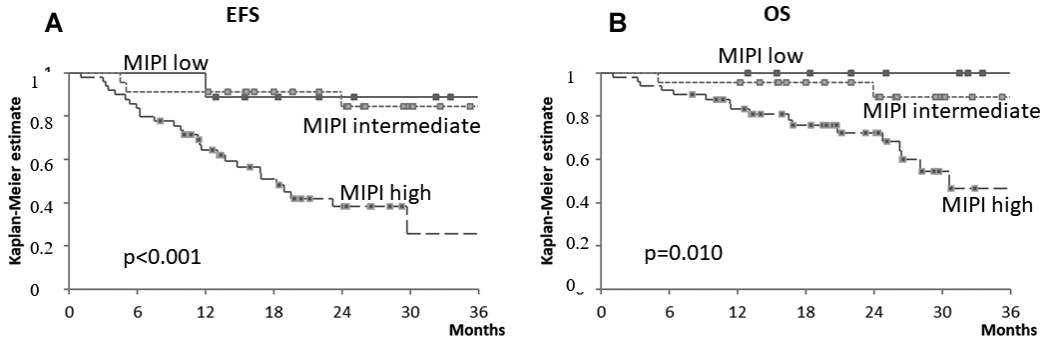
Event-free survival (EFS) (**A**) and overall survival (OS) (**B**) of patients according to mantle cell international prognostic index (MIPI).

**Figure 8:**
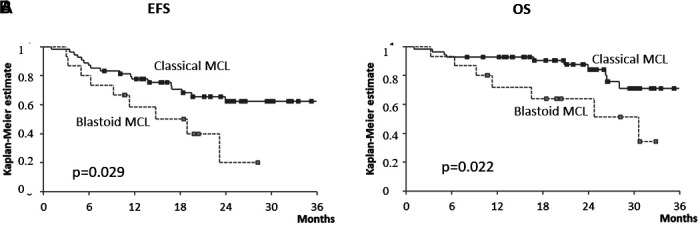
Event-free survival (EFS) (**A**) and overall survival (OS) (**B**) according to morphology. Full line – classical; dashed line – blastoid.

**Figure 9:**
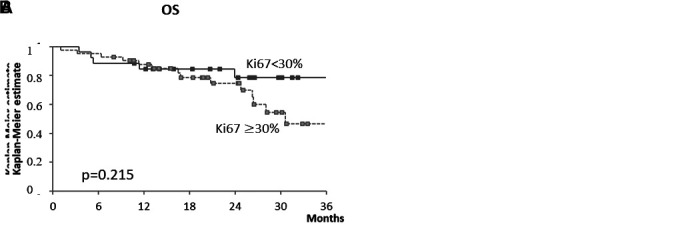
Event-free survival (EFS) (**A**) and overall survival (OS) (**B**) according to Ki67 expression. Full line – Ki67 < 30%; dashed line – Ki67 ≥ 30%.

Biologic prognostic factors (MIPI, morphology, and Ki67) were analyzed only in the recent cohort, as these data were not available for the historical cohort. Patients with a high MIPI had significantly inferior EFS and OS compared with those with an intermediate and low MIPI (Figure 7). Patients with an intermediate and low MIPI did not differ in the outcomes. Patients with blastoid MCL had inferior EFS and OS compared with those with classical MCL (Figure 8). Ki67 expression did not significantly affect the outcomes (Figure 9). The effect of the examined biological factors was independent of age or treatment (data not shown).

## DISCUSSION

Our study suggests that two-year EFS (17%) and two-year OS (26%) in the recent cohort improved significantly compared with the historical cohort. The observed improvement in patients able to tolerate aggressive treatment approaches may be explained by the use of HD-AraC-based induction regimens and ASCT in the first remission, the latter especially in patients treated with R-CHOP. This is in accordance with North American and Australian series (7,8), which also showed that ASCT was more beneficial for the subgroup of patients who did not receive HD-AraC-based induction. These findings cast doubt on the benefit of ASCT in the first remission in patients receiving more intensive induction; clinical trials addressing this question are under way. Regarding non-transplantable patients, our results are in accordance with a seminal randomized trial showing the superiority of BR over R-CHOP (9) and data from the UK (10). However, the outcomes of these patients improved less. This population might in the future benefit from the introduction into front-line treatment of new agents, such as ibrutinib and other Bruton tyrosine kinase inhibitors. With rituximab maintenance, we observed improved EFS, but not OS. Other real-life studies found improvement in both EFS and OS (7,10,11). The lack of effect on OS in our study might be due to short follow-up, but in at least one real-life study maintenance influenced neither OS nor EFS (8). In all comparisons between the recent and historical cohort that we performed, the differences in OS were more pronounced than those in EFS, probably as a consequence of improvements in the treatment of relapsed/refractory disease, eg, the introduction of ibrutinib treatment. The outcomes of our patients treated with specific regimens and/or ASCT are similar to those of equivalently treated patients from population-based, real-life cohorts from the UK, USA, and Australia (7,8,10).

The Croatian Society for Hematology and KroHem have since 2006 published and regularly updated recommendations for the diagnosis and treatment of lymphomas (12,13). We believe that this helped to achieve the favorable results published in our study. Our opinion is supported by the fact that the results in both cohorts seem superior to the results of contemporaneous patients in the UK, a significant number of whom were treated with chlorambucil with/without rituximab and only 8% of patients were autografted in the first remission (10).

Five (6%) newly diagnosed patients were deemed indolent and not treated; this number is somewhat lower than in other series (14). None of these patients required therapy during the follow-up, confirming that patients with MCL with limited tumor burden and no symptoms can safely be observed without initiating treatment.

Blastoid morphology and high MIPI remain important negative prognostic factors irrespective of the front-line therapy. This is equivalent to the results of other population-based studies analyzing biologic prognostic factors (7,8,11). The lack of difference in outcomes between patients with low and intermediate MIPI might be a consequence of the low number of treated patients with very favorable disease characteristics in our study, but although not always stated explicitly, seems also to have been noted in other real-life studies. Ki67 was not of prognostic significance in our study, in contrast to the UK and Australian experience (8,10). This is possibly related to problems of expression quantification in this tumor type (15).

The main weaknesses of our study are related to the relatively short follow-up of the recent cohort and possible bias in data collection. Still, demographic characteristics of both cohorts are very similar, suggesting that they are comparable. There were 575 new cases of NHL in 2015 in Croatia (16). Based on our results, there should be about 30 newly diagnosed patients with MCL annually in Croatia, comprising 5% of all NHL cases. This is within the 3% to 10% range described in the literature. Furthermore, due to the retrospective design of the study, the presented data and *P* values should be considered mostly as descriptive values.

In conclusion, our study suggests that treatment changes in patients with MCL in the last decade significantly improved the EFS and OS. The use of HD-AraC-containing induction regimens and ASCT in the first remission seem most important for patients able to tolerate aggressive therapies, while BR induction benefits non-transplantable patients. Rituximab maintenance also improves EFS. Finally, our experience shows that communication between peers and evidence-based and regularly updated national recommendations can significantly improve the outcomes of patients with lymphomas, even without the broad use of new expensive agents.
